# High Selective Mixed Membranes Based on Mesoporous MCM-41 and MCM-41-NH_2_ Particles in a Polysulfone Matrix

**DOI:** 10.3389/fchem.2019.00332

**Published:** 2019-06-17

**Authors:** Marius Gheorghe Miricioiu, Ciprian Iacob, Gheorghe Nechifor, Violeta-Carolina Niculescu

**Affiliations:** ^1^National Research and Development Institute for Cryogenics and Isotopic Technologies - ICSI Ramnicu Valcea, Ramnicu Valcea, Romania; ^2^Department of Analytical Chemistry and Instrumental Analysis, Politehnica University of Bucharest, Bucharest, Romania

**Keywords:** gas separation, mesoporous silica (MCM-41), mixed matrix membrane (MMM), nanoparticles, polysulfone

## Abstract

The development of membrane technology for gas separation processes evolved with the fabrication of so-called mixed matrix membranes (MMMs) as an alternative to neat polymers, in order to improve the overall membrane effectiveness. Once the mixed matrix membranes are used, the gas separation properties of the porous materials used as fillers are combined with the economical processability and desirable mechanical properties of polymer matrix. Mixed mesoporous silica/polymer membranes with high CO_2_ and O_2_ permeability and selectivity were designed and prepared by incorporating MCM-41 particles into a polymer matrix. Ordered mesoporous silica MCM-41 with high surface confirmed by BET analysis were obtained and functionalized with amino groups. In order to obtain the mixed membranes, the mesoporous silica was embedded into the polysulfone matrix (PSF). Flat mixed matrix membranes with 5, 10, and 20 wt% MCM-41 and MCM-41-NH_2_ loadings have been prepared via the polymer solution casting method. The phase's interactions were studied using scanning electron microscopy (SEM), X-ray diffraction (XRD), Fourier transformed infrared spectroscopy (FTIR) and thermogravimetry (TGA), while the gas separation performances were evaluated using pure gases (CO_2_, O_2_, N_2_). The MCM-41/PSF and MCM-41-NH_2_/PSF membranes exhibited increased permeabilities for O_2_ (between 1.2 and 1.7 Barrer) and CO_2_ (between 4.2 and 8.1 Barrer) compared to the neat membrane (0.8 Barrer). The loss of selectivity for the O_2_/N_2_ (between 6 and 8%) and CO_2_/N_2_ (between 25 and 41%) gas pairs was not significant compared with the pure membrane (8 and 39%, respectively). The MCM-41/PSF membranes were more selective for CO_2_/N_2_ than the O_2_/N_2_ pair, due to the size difference between CO_2_ and N_2_ molecules and to the condensability of CO_2_, leading to an increase of solubility. Stronger interactions have been noticed for MCM-41-NH_2_/PSF membranes due to the amino groups, with the selectivity increasing for both gas pairs compared with the MCM-41/PSF membranes.

## Introduction

Membrane-based gas separation processes are relatively new technologies and have received significant attention due to their main advantages, such as being environmentally friendly, simplicity, and low operating cost (Zornoza et al., 2009, 2013; Li et al., 2012; Yu et al., 2012; Zhang et al., 2013; Rezakazemi et al., 2014). These advantages are not enough if the membrane fabrication is laborious and costly, as in the case of inorganic membranes. Therefore, the mixed membranes could be a viable solution for the conventional separation processes (pressure swing adsorption, thermal swing adsorption and cryogenic distillation) once the drawbacks are overcome.

The polymeric membranes were intensively studied but other limitations appeared, such as the trade-off between the permeability and the selectivity of the membrane, according to Robeson curves (Kim and Marand, 2008; Robeson, 2008). Taking this aspect into account, the glassy polymer membranes are characterized by high selectivity and low permeability, in comparison to rubbery polymer membranes which present high permeability and low selectivity (Rezakazemi et al., 2014).

The development of the ideal membranes (defect-free membranes), suitable for gas separation processes, at reasonable costs, remains a challenge that can possibly be overcome by the synthesis of so-called mixed matrix membranes. These are an alternative to commercial neat polymeric membranes and also to inorganic membranes, by combining their advantages, such as the easy processability of the polymers, with high gas separation properties of the fillers (Radu et al., 2014).

The separation properties of the membranes are directly dependent on both the pore structure of the materials embedded in the polymer and the interaction of the two phases, filler-polymer (organic-inorganic) (Roman et al., 2007; Hamid and Jeong, 2018). This can lead to an increase or decrease of the selectivity by some phenomena in the membrane, such as the plasticization or the non-selective void appearance. Therefore, it is very important to combine two compatible phases. Various fillers including zeolites (Shen and Lua, 2012; Rostamizadeh et al., 2013; Barquin et al., 2016), silica (Merkel et al., 2002; Jomekian et al., 2011; Zanoletti et al., 2018), carbon molecular sieves (Anson et al., 2004; Rafizah and Ismail, 2008; Weng et al., 2010), carbon nanotubes (Majeed et al., 2012; Khan et al., 2013; Nour et al., 2013; Ahnmad et al., 2014), and metal organic frameworks (Basu et al., 2011; Shahid and Nijmeijer, 2017) were used for the fabrication of mixed matrix membranes by their embedding into different polymers, such as cellulose acetate, polysulfone, polyimide, polyamide, polyphenylene oxide, polycarbonate, and polydimethylsiloxane. Not all of these materials proved to be a good option. Poor interfacial adhesion was reported between zeolite 4A and glassy polymer (Mahajan and Koros, 2000, 2002a,b; Yong et al., 2001; Moore and Koros, 2005) and the same behavior was also obtained in the case of the carbon nanotubes (Ma et al., 2010; Sears et al., 2010). Some studies reported a strong affinity between metal organic frameworks and polymer matrix, avoiding the non-selective gap (Gascon et al., 2012; Zornoza et al., 2013; Rezakazemi et al., 2014), producing plastic deformation of the matrix by elongation of the polymer (Perez et al., 2009). The mesoporous silica materials, such as MCM-41 or MCM-48, due to their pore diameters (2–5 nm) and high surface area, were considered good candidates for the fabrication of mixed matrix membranes (Ravikovitch and Neimark, 2000; Schumacher et al., 2000; Zornoza et al., 2009; Zhao et al., 2016).

Generally, the membranes containing mesoporous silica as filler can be produced by three methods: (a) direct mixing/blending of the silica nanoparticles into the polymer matrix, (b) a sol–gel method in which the silica nanoparticles can be synthesized *in situ* in the presence of a preformed organic polymer, and (c) *in situ* polymerization involving the dispersion of the silica in the monomer before the polymerization is carried out (Chen et al., 2016). In this work, the first method was adopted after obtaining the silica nanoparticles by a sol-gel synthesis. Modification of the mesoporous materials with organic groups is required to enhance their specific sorption capacities, and effective methods for functionalization with appropriate modification agents are crucial for advancing their practical application (Kim et al., 2015a).

The main goal of this study was the development of mixed matrix membranes based on mesoporous silica and polysulfone (PSF) polymer and the demonstration of their higher separation performances toward the neat membranes. Polysulfone was selected as the membrane matrix due to its permeability-selectivity combination close to Robeson's “upper bound” region (Robeson, 2008). Furthermore, PSF has been previously used as a matrix for zeolite composite membranes (Duval et al., 1994; Suer et al., 1994; Battal et al., 1995; Hamid and Jeong, 2018). The preparation and characterization of the mixed membranes is described for several mesoporous silica loadings. Additionally, the permeabilities of N_2_, O_2_ and CO_2_ at ambient temperature were determined.

## Materials and Methods

### Synthesis of Mesoporous Silica Material—MCM-41

The MCM-41 mesoporous silica particles were synthesized using hexadecyltrimethylmonium bromide, tetramethyl ammonium hydroxide and sodium silicate (Sigma-Aldrich - Steinheim, Germany), in accordance with the procedure reported in the literature (Niculescu et al., 2012). Shortly, 5.6 mg of hexadecyltrimethylmonium bromide was dispersed in 60 g ultrapure H_2_O (Purelab Flex 3 - Elga, Wycombe, United Kingdom), stirring the mixture for 2 h at room temperature. A total of 7.6 g sodium silicate was then added, stirring the mixture for 2 h. After this period, 43.36 g tetramethyl ammonium hydroxide was added, with stirring for 30 min. The pH was adjusted to 10.5 and was checked after 15 min. The mixture was stirred for 24 h, with the pH being again checked, and then the mixture was introduced into an autoclave at 100°C for 5 days. The resulting mixture was filtered under vacuum, washed with water and dried, then it was calcined at 600°C.

### MCM-41 Functionalization

The amino functionalized silica was obtained by activating MCM-41 overnight under vacuum, then treating MCM-41 with anhydrous toluene under stirring (Niculescu et al., 2011). Over the resulting solution, 3-triethoxysilylpropylamine (Sigma Aldrich - Steinheim, Germany) was added dropwise under continuous stirring, and the mixture was kept under reflux for 5 h at 120°C. After the functionalized silica was formed, the mixture was filtered, washed and it was subjected to a continuous extraction using diethyl ether / dichloromethane in a Soxhlet apparatus (Sigma Aldrich - Steinheim, Germany) and dried at room temperature.

### Mixed Matrix Membrane Preparation

The mixed matrix membranes (PSF/MCM-41) were obtained from polysulfone (PSF) pellets (Sigma-Aldrich - Steinheim, Germany) via the solution casting method (Zornoza et al., 2009; Murali et al., 2014). Prior to the membrane synthesis, the PSF was conditioned at 105°C for 3 h, under vacuum, in order to remove the adsorbed water, and then three main steps were performed to accomplish the membrane fabrication.

The first step consisted of the dispersion of various quantities of MCM-41 in chloroform (Sigma-Aldrich - Steinheim, Germany) using an ultrasonic bath (Elma S60H - Elma Schmidbauer GmbH, Singen, Germany) for 20 min, in order to obtain mixed matrix membranes with 5, 10, and 20 wt.% MCM-41 as filler. In the second step, the PSF was added to the obtained solution, and the mixture was magnetically stirred for 24 h in order to obtain a homogeneous membrane. During the stirring process, five sonication intervals were performed to enable the penetration of the MCM-41 pores by the polymeric chain.

In the last step, the mixtures were cast in Petri glass dishes and left overnight, at room temperature, partially closed to slow the natural evaporation of solvent. The membranes were removed by flushing the plates with ultrapure water and then were dried in the vacuum oven at 110°C and 80 mbar for 24 h.

Neat membranes, formed only from PSF, were prepared using the same recipe in order to compare them with the mixed membranes.

The thicknesses of the obtained membranes were determined by using a digital micrometer (Schut Geometrische Meettechniek - Groningen, Netherlands) with ± 0.001 mm accuracy for the 0–25 mm measurement interval. A membrane with a 5 cm^2^ diameter was cut from the obtained material.

### Membranes and Materials Characterization

The MCM-41 specific area was determined by using the Brunauer-Emmett-Teller (BET) and Barrett-Joyner-Halenda (BJH) methods. The effects of MCM-41 loadings were investigated by scanning electron microscopy (SEM), thermogravimetric analysis (TG) and single gas (pure O_2_, N_2_ and CO_2_) permeability measurements.

The IR spectra were recorded in the region 4000–400 cm^−1^ on a CARY 630 instrument (Agilent Technologies - Santa Clara, CA, USA) in anhydrous KBr pellets. Before the analysis, both KBr and samples were grinded in an agate mortar and pestle, then dried at 80°C under vacuum for 3 h in order to avoid the appearance of physically adsorbed water.

The scanning electron microscopy images were collected on a Variable Pressure Field Emission Scanning Electron Microscope, FESEM VP (Carl Zeiss - Oberkochen, Germany), with a resolution of 0.8 nm at 30 kV or 2.5 nm at 30 kV in VP mode.

Transmission electron microscopy measurements were performed on Tecnai G2 F30S-TWIN (Thermo Fisher Scientific former FEI, Eindhoven, Netherlands), equipped with a STEM/HAADF detector, EDS (Energy dispersive X-ray Analysis and EFTEM, EELS (Electron energy loss spectroscopy)). The microscope was operated at an acceleration voltage of 300 KV. In order to prepare the sample, a small amount of powder was dispersed into deionized water and sonicated for 15 min. After that 10 μL diluted sample was placed onto a 400-mesh holey carbon-coated Cu grid. The sample was left to dry fully before the TEM investigations.

Thermogravimetric analysis was performed using a SDT Q600 V20.5 Build 15 instrument (TA Instruments - New Castle, DE, USA). The weight changes during the heat treatment of the MCM-41 and PSF/MCM-41 membranes were evaluated under N_2_ atmosphere (flow rate: 100 mL/min, 99.999% vol purity) with a heating rate of 10°C/min in the 30–1000°C range.

X-ray diffraction analysis was carried out on a Rigaku Ultima IV X-ray diffractometer equipped with a CuKα source (Rigaku Co. – Tokio, Japan). The measurements were carried out within the range of 1° ≤ 2θ ≤ 8° with a step increment ratio of 0.02°/2 s for MCM-41 powder.

Brunauer-Emmett-Teller (BET) specific surface area, N_2_ isotherm and Barrett-Joyner-Halenda (BJH) pore size distribution were obtained for the mesoporous silica with a Quantachrome Autosorb-IQ porosity analyzer (Quantachrome Instruments - Boynton Beach, FL, USA). The MCM-41 was outgassed at 150°C to the measurements, whereas the functionalized silica was outgassed at 120°C. The N_2_ adsorption and desorption were measured at −196°C.

### Gas Permeability Measurements

**Figure 1:**
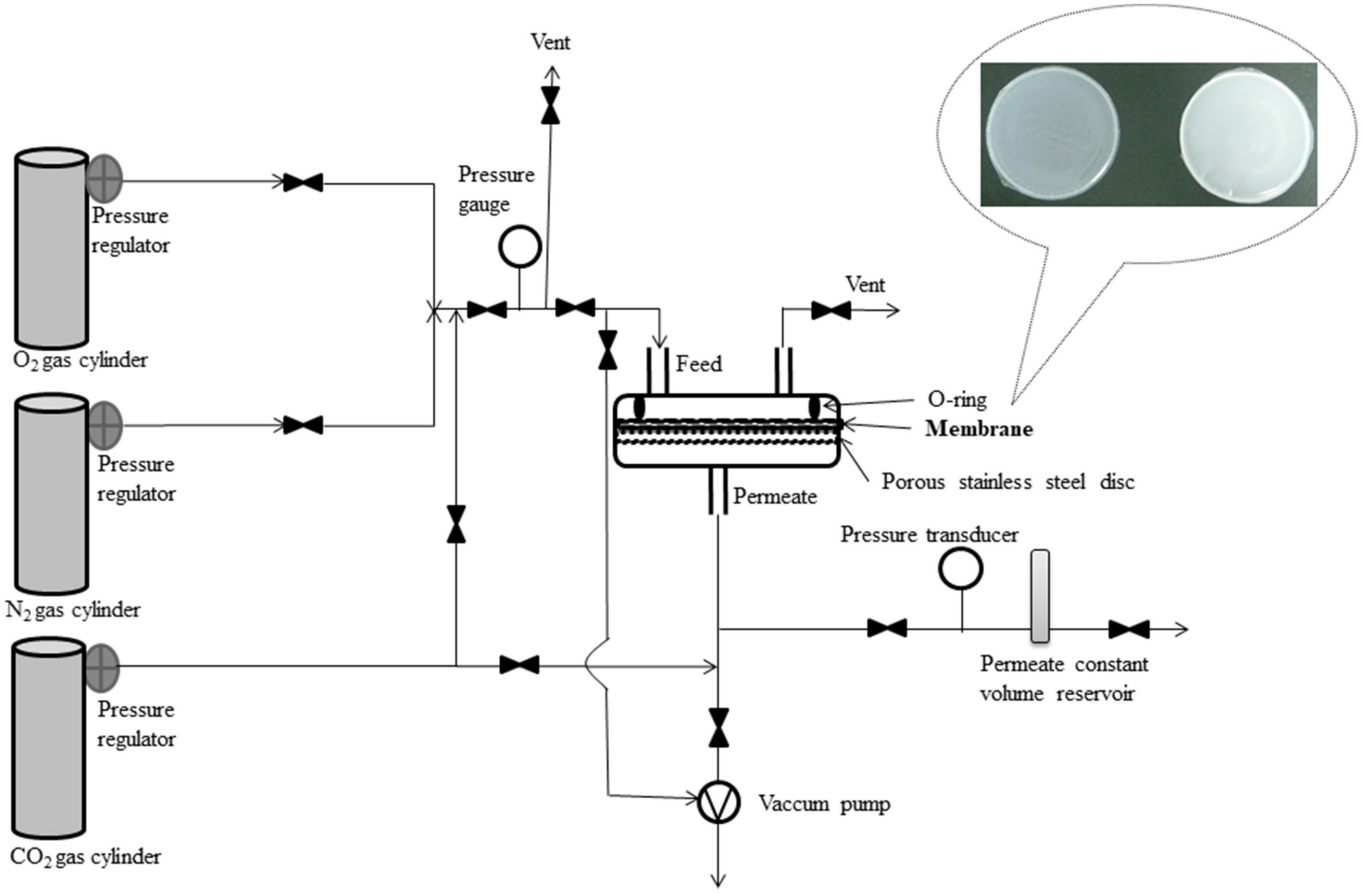
Experimental setup for testing the membranes performances.

The pure gas permeability measurements were carried out at room temperature using a constant volume-variable pressure system similar to those described in the literature (Koros et al., 1977; Felder and Huvard, 1980; Lin et al., 2000; Barquin et al., 2016) and schematically presented in Figure 1.

The membrane cell consisted of two stainless steel pieces with a cavity where the membrane sample was placed on a porous disk support of 25 μm nominal pore size (Mott Corp., Farmington, CT, USA) and sealed with a Viton O-ring in order to reach high pressure. One of the cell pieces represented the upstream side or the gas feed side and the other was downstream side or the permeate side. The pressure from the inlet side of the membrane cell was kept constant, at 1 bar, while the accumulated gas pressure increase in the permeate side was measured using a transducer (Omega, Manchester, UK) and plotted vs. time. Before each permeability experiment, the membrane was exposed to vacuum, for 10 h, using an oil-free pump (KNF Laboport vacuum pump – Sigma-Aldrich - Steinheim, Germany). Each gas was passed through each type of membrane five times, and the average of the results was used for data interpretation.

The gas permeability coefficient was calculated using the following equation:

(1) $$\begin{array}{l} \text{P}=\frac{273\times 10^{10}}{760}\;\frac{\text{VL}}{\text{AT}(\frac{\text{p}{\;}_{0}\times 76}{14.7})}\;\frac{\text{dp}}{\text{dt}} \end{array}$$

where P is the gas permeability represented in Barrer (1 Barrer = 1 × 10^−10^ cm^3^ (STP) cm/cm^2^ s cm Hg), V is the permeating gas volume (cm^3^), L is the membrane thickness (cm), A is the membrane area (cm^2^), T is the experimental temperature (K), p_0_ is the feed gas pressure (psia) and dp/dt is the pressure rate measured by the pressure sensor in the downstream chamber (mmHg/s).

The ideal selectivity was determined from the equation:

(2) $$\begin{array}{l} \propto =\frac{P_{A}}{P_{B}} \end{array}$$

where P_A_ and P_B_ are the permeabilities of the pure gases A and B.

## Results and Discussion

**Figure 2:**
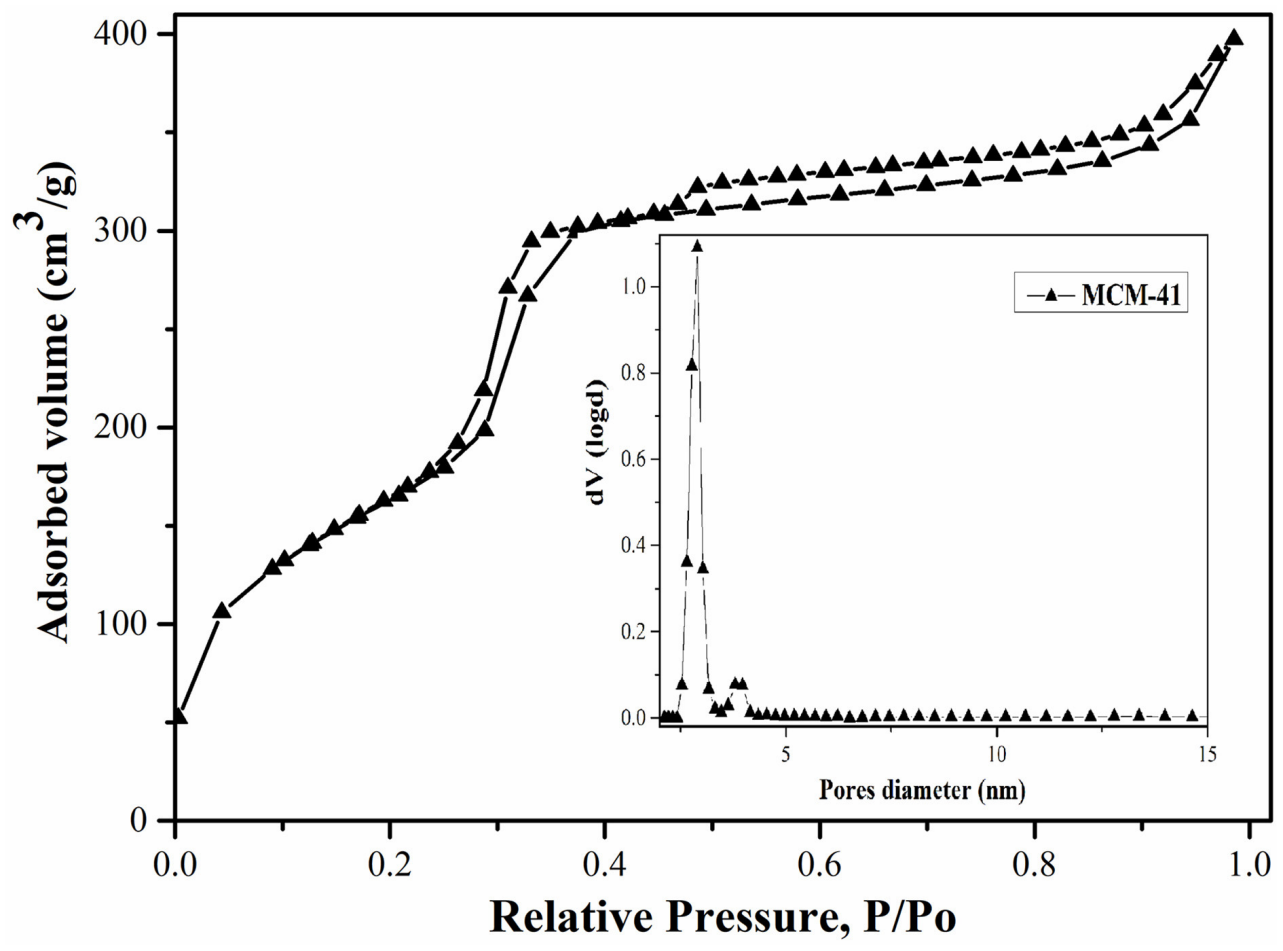
MCM-41 adsorption isotherm and pores distribution.

**Figure 3:**
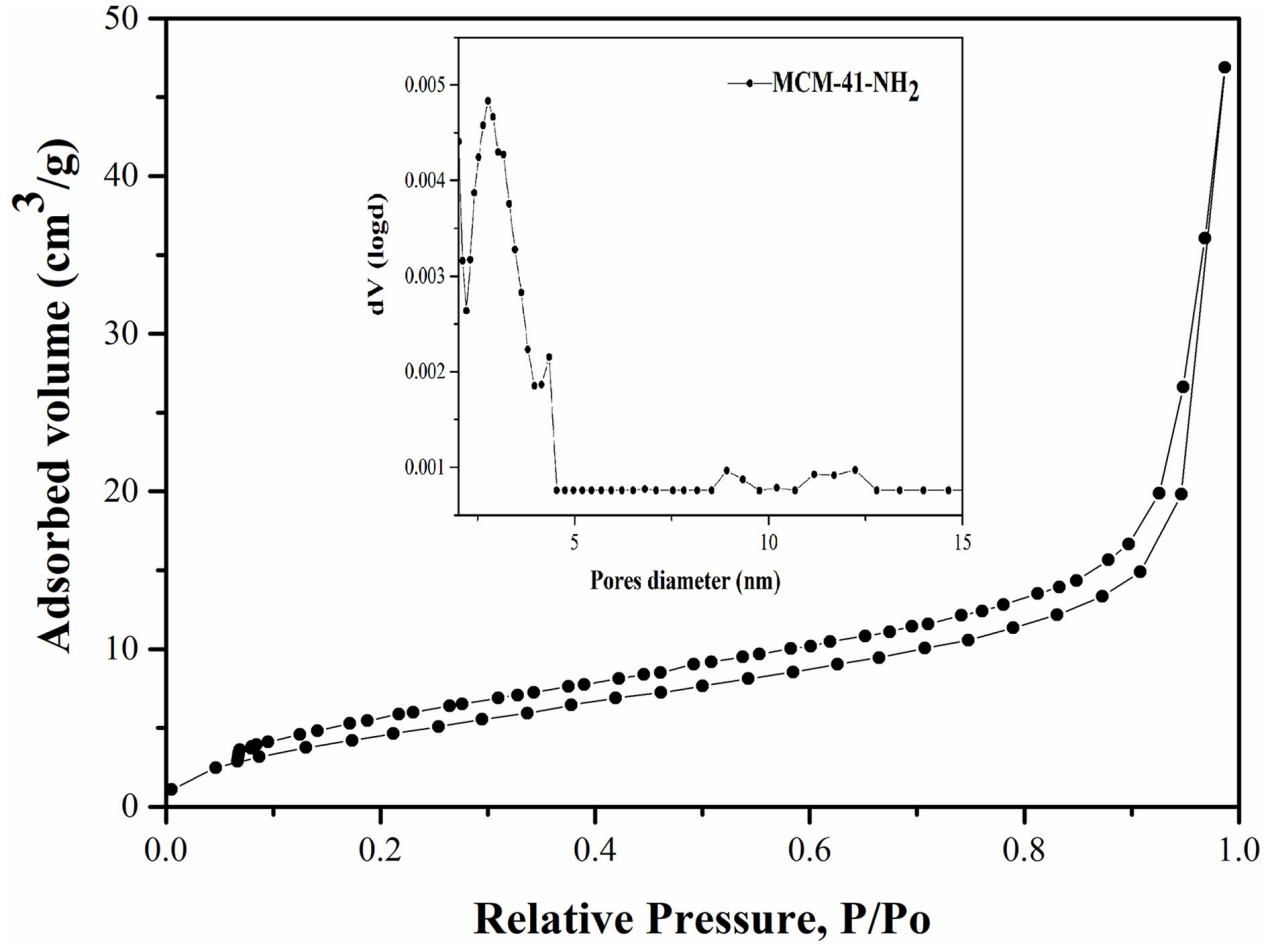
MCM-41-NH_2_ adsorption isotherm and pores distribution.

N_2_ adsorption-desorption isotherms (Figures 2, 3), obtained at 77 K, correspond to irreversible type IV isotherm as defined by IUPAC. The BET surface area for MCM-41 was 1160 m^2^/g, indicating a high-quality material. The BJH investigations revealed an average pore diameter of 3.2 nm (Figure 2).

The functionalization of MCM-41 led to a drastic decrease of the specific surface area to 12 m^2^/g due to the blocking process with the aminopropyl groups (Figure 3). Also, it was observed that the pore diameters remain approximately in the same range (Figure 3), demonstrating that the mesoporous structure was kept after functionalization. Although the specific surface area decreased, the ordered mesostructure was not collapsed. This fact was confirmed by adsorption-desorption measurements, which showed that pore diameters remain approximately in the same range in the case of MCM-41-NH_2_ as in the case of MCM-41 (Figures 2, 3). The MCM-41 presented an average pore diameter of 3 nm and the MCM-41-NH_2_ presented an average pore diameter of 2.8 nm. The decrease of BET surface area, total pore volume and average pore diameter indicated the presence of aminopropyl functional group at the MCM-41 surface.

The presence of mesopores after functionalization allows facile access for other reagents, being very important in environmental applications.

**Figure 4:**
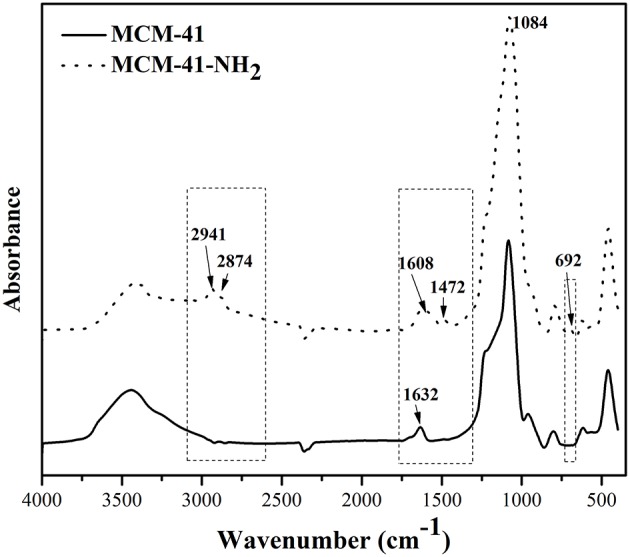
FTIR spectra of mesoporous filler.

The mesoporous silica functionalization was confirmed through FTIR spectroscopy. Figure 4 presents the IR spectra for MCM-41 and functionalized MCM-41.

In the region 3435 cm^−1^, MCM-41 presents specific band characteristic for the OH groups from the silanol surface or to the physical adsorbed water from the surface. After functionalization with 3-triethoxysilylpropylamine, the intensity of this band decreases at the same time as the appearance of some band characteristic to immobilized amines from aminopropyl groups of 3-triethoxysilylpropylamine, indicating that the OH groups from the initial matrix reacted with ethoxy-groups of the organic precursor. The functionalized silica presents two bands of medium intensity at 2941 cm^−1^ and 2874 cm^−1^, characteristic for 3-triethoxysilylpropylamine—these bands being absent in the initial porous material. Also, for the functionalized samples, a medium intensity band at 1627 cm^−1^ was observed, which was attributed to δ_NH2_. These results confirm, one more time, the functionalization of the porous material with aminopropyl organic function. The bands from 1084 cm^−1^ and 692 cm^−1^ were attributed to Si-O-Si and Si-O vibrations (Liang et al., 2009; Hoang et al., 2010). The absorption bands from 1632 cm^−1^ of MCM-41-NH_2_ and from 1608 cm^−1^ of MCM-41 can be attributed to stretching vibrations of adsorbed water molecules (δ_H−O−H_). The bands from 940 cm^−1^in the MCM-41 spectrum were attributed to Si-OH stretching (Liang et al., 2009).

**Figure 5:**
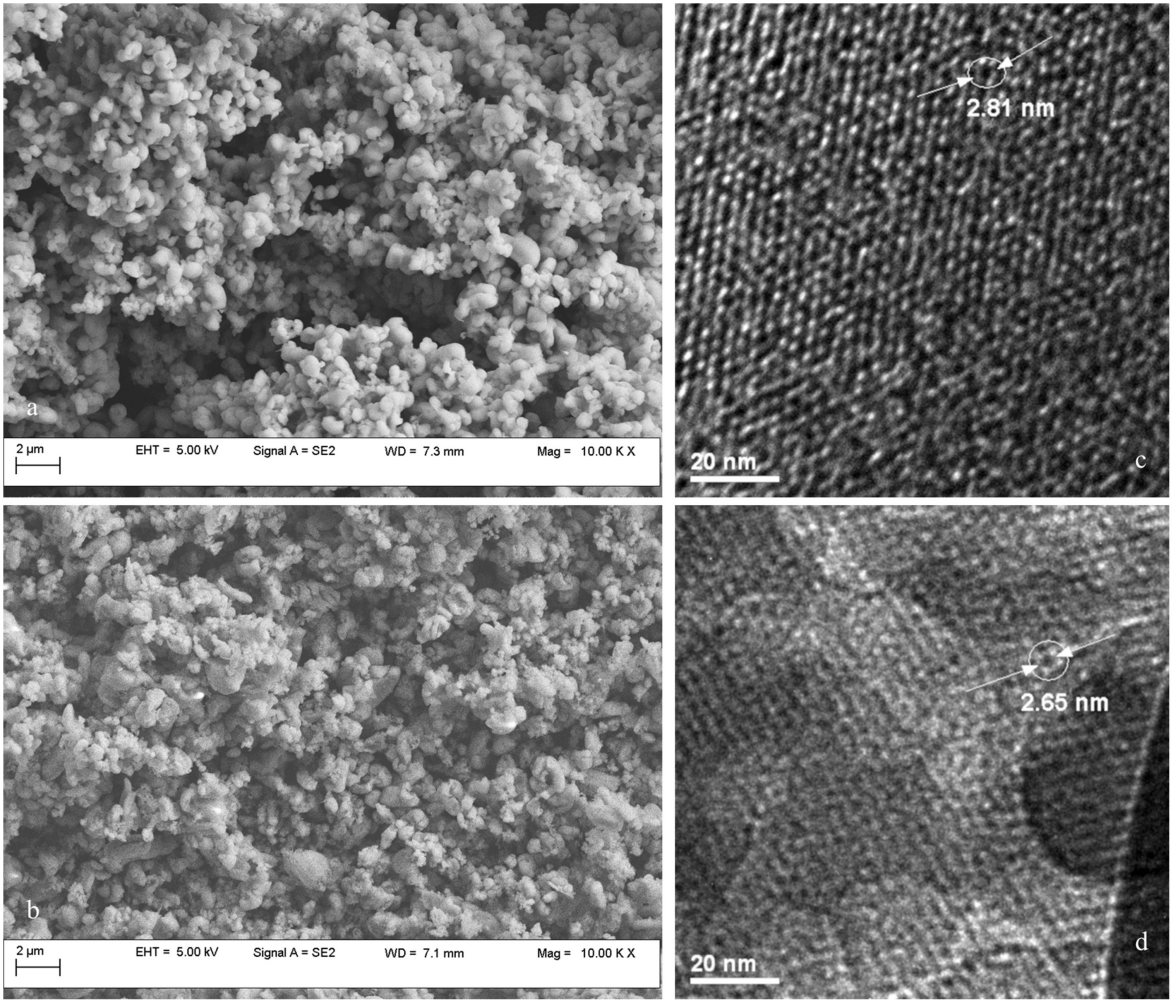
SEM and TEM images for MCM-41 **(a,c)** and MCM-41-NH_2_ **(b,d)**.

The morphology of synthesized MCM-41 and MCM-41-NH_2_ mesoporous silica was examined by scanning electron microscopy (SEM) operated at a voltage of 5 kV. The samples were attached to aluminum stubs with double side adhesive carbon tape. MCM-41 samples exhibit regular sphere-shaped particles, having smooth surface morphology (Figure 5a). Functionalization alters the spherical shape and yields agglomerated nanoparticles (Figure 5b).

The TEM images (Figures 5c,d) evidenced the characteristic pore arrangement, a honeycomb-like structure of the MCM-41. The ordered hexagonal nano-channels throughout the mesoporous material indicate good homogeneity (Chenite et al., 1995). TEM micrograph (Figures 5c,d) confirms that the MCM-41-NH_2_ contains well-ordered, two-dimensional porous structure, similar to MCM-41. Furthermore, pore diameter after functionalization of MCM-41 was found to be 2.65 nm (Figure 5d), which is in accordance with the value of 2.7 nm computed by the BJH analysis. The decrease in pore diameter is due to the presence of aminopropyl groups grafted to MCM-41 inner walls.

**Figure 6:**
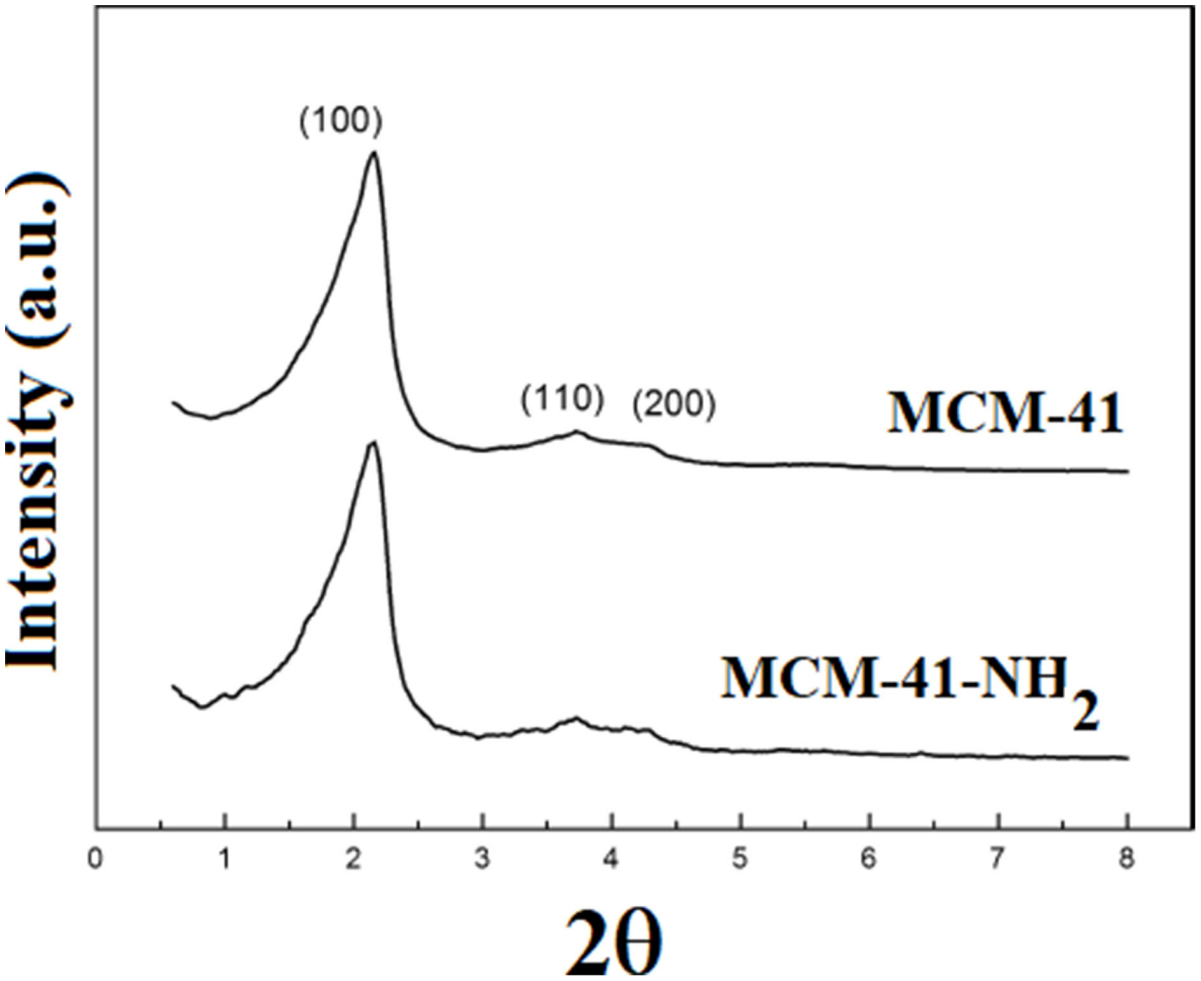
XRD for MCM-41 and MCM-41-NH_2_.

Figure 6 presents the X-ray diffraction pattern of the mesoporous silica before and after functionalization. The XRD pattern of the MCM-41 consisted of a typical reflection at 2.19° and weak overlapped reflections at 3.92° and 4.50°, corresponding to (100), (110), and (210) planes of MCM-41, suggesting a hexagonal mesoporous silica structure (Luo et al., 2016). In the X-ray diffraction pattern of MCM-41-NH_2_, the presence of three lines can be noticed: 2θ = 2.20° (100), 2θ = 3.81° (110), and 2θ = 4.48° (210), which are also presented in MCM-41 spectra.

**Figure 7:**
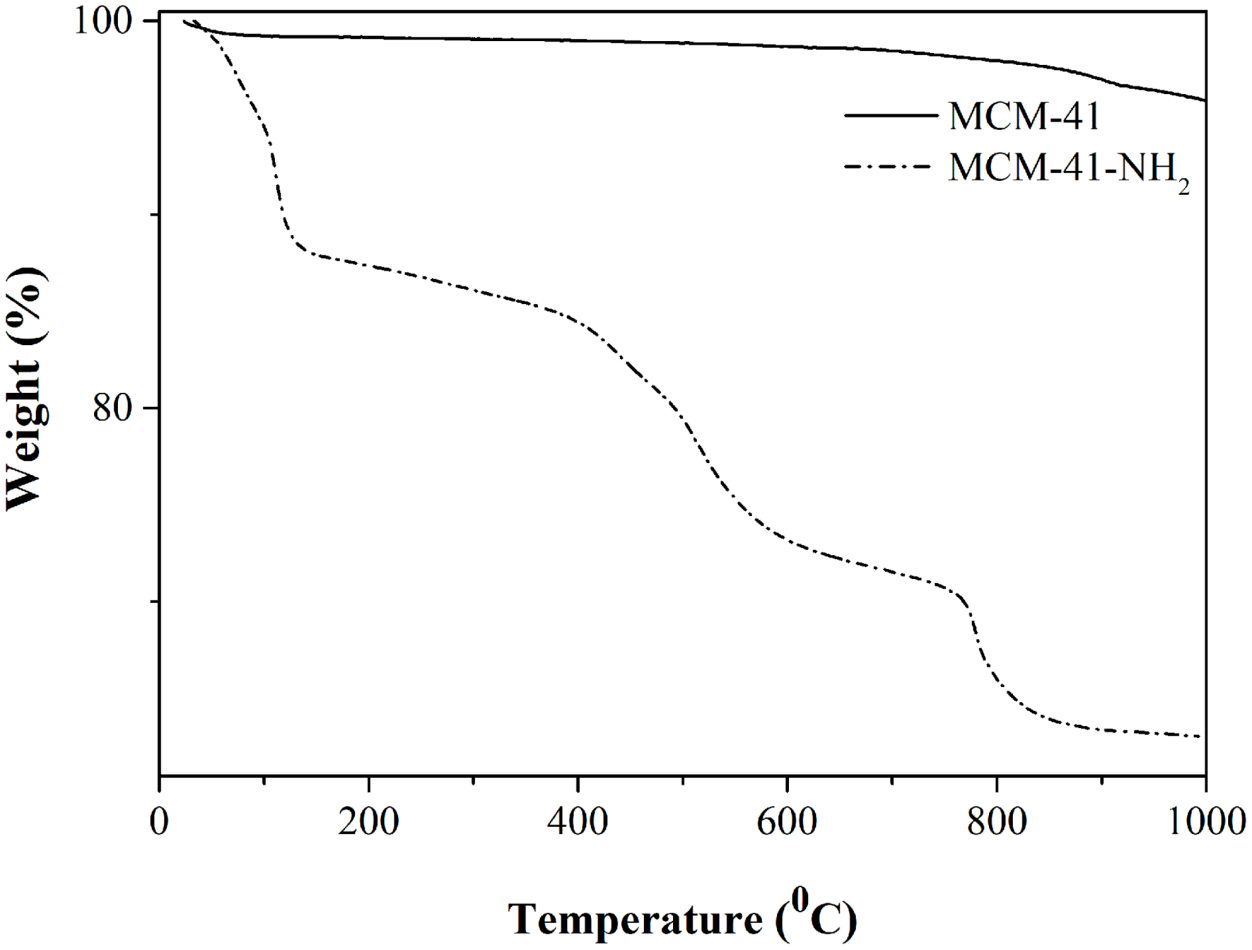
TG analysis of MCM-41 and MCM-41-NH_2_.

TG analysis of MCM-41 (Figure 7) highlighted two intervals of weight loss, the first caused by desorption of the water linked to the silica surface, the second one being attributed to the mesoporous structure disruption. In the case of MCM-41-NH_2_ (Figure 7), three intervals of weight loss were visible: 30–150°C, attributed to water loss; 300–600°C, attributed to the fragmentation of the APTES attached to the MCM-41 surface; and > 600°C, attributed to the disruption of the rest of the mesoporous structure (Mello et al., 2011; Saad et al., 2016). The total mass loss for MCM-41 and MCM-41-NH_2_ was about 4 and 37%, respectively.

The obtained mixed matrix membranes had a thickness of around 30 μm, mediating the measurements of ten different points from each membrane.

Poor wetting properties between the polymer and the mesoporous silica filler may lead to non-selective void formation or to the inorganic particles agglomeration in the polymer, resulting in the loss of the membrane selectivity or mechanical properties. In order to investigate the dispersion of the mesoporous silica in the PSF matrix, the SEM images were inspected. Prior preparation of the membranes' samples was required in order to assure the electron conductivity and to protect the membranes from damage. First, the membranes were immersed into liquid nitrogen and fractured, then they were covered with a thin gold layer (~20 nm), obtaining the cross-section (a, c, e) and plane (b, d, f) images.

**Figure 8:**
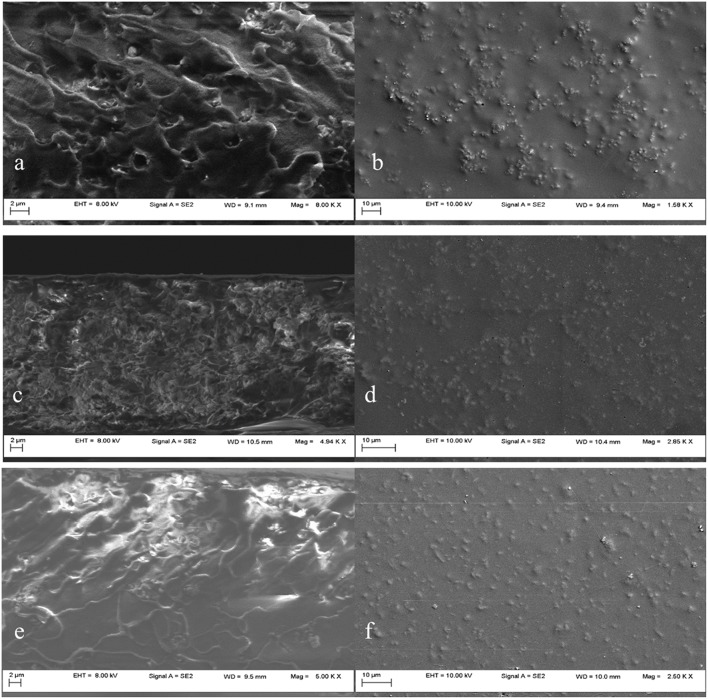
SEM images of MCM-41/PSF membranes with various loadings: 5 wt% MCM-41 **(a,b)**; 10 wt% MCM-41 **(c,d)**; 20 wt% MCM-41 **(e,f)**.

SEM cross-section and plane images of 5, 10 and 20 wt% unmodified MCM-41/PSF mixed matrix membranes are shown in Figure 8. Figures 8a,c,e shows that unmodified MCM-41 silica particles agglomerated and formed micrometer-scale void spaces between the polymer matrix and the silica phase. Instead, the plane sections from Figures 8b,d,f show a good dispersion of the mesoporous silica filler in the polymer matrix. The unmodified mesoporous silica has a high surface area covered by hydrophilic silanol groups. Consequently, the silica particles easily adhere to each other via hydrogen bonding (Sun et al., 2005).

The SEM results of 10 wt% MCM-41 membranes, shown in Figures 8c,d, are similar to those with 5 wt% membranes. Even at a loading of 20 wt %, as shown in Figures 8e,f, the silica nanoparticles are still well dispersed throughout the polymer matrix. Some agglomerations of particles were visible for the 20 wt% MCM-41/PSF membrane (Figure 8c), but not significant, due to the hydrogen bonding tendency. The resulting composites are free-standing films which hold up to gas permeability measurements. The penetration of polymer chains into the MCM-41 pores was possible due to the 3.2 nm average pore diameter, confirmed by MCM-41 pore size distribution analysis.

**Figure 9:**
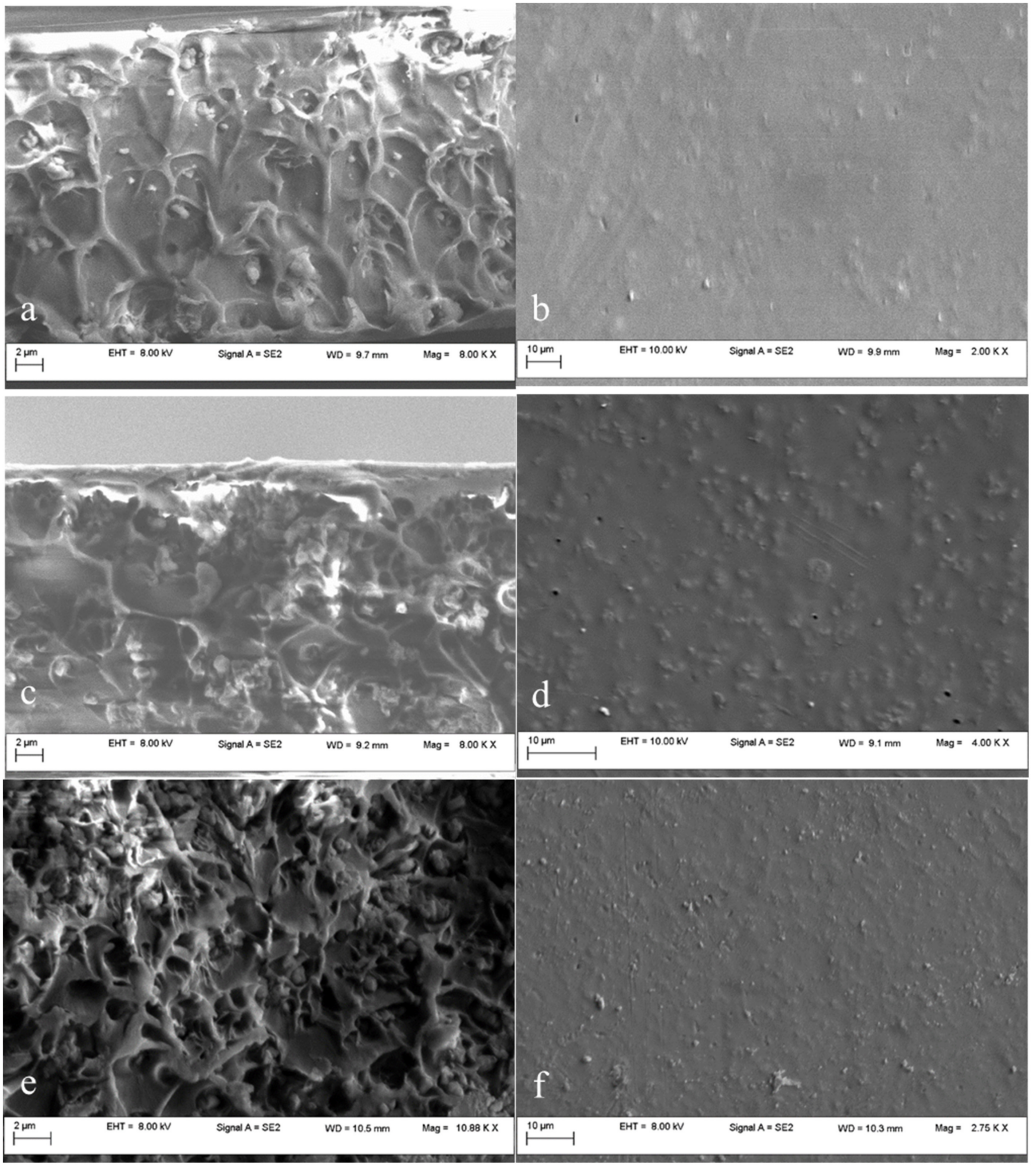
SEM images of MCM-41-NH_2_/PSF membranes with various loadings: 5 wt% MCM-41-NH_2_ **(a,b)**; 10 wt% MCM-41-NH_2_ **(c,d)**; 20 wt% MCM-41-NH_2_ **(e,f)**.

Functionalized mesoporous silica particles, as shown in Figure 9(a,c,e-cross section,b,d f-plane) appear to be better dispersed throughout the polymer matrix. After the modification of the silica with the aminopropyl groups, the external hydrophilic surface is changed into a hydrophobic surface. This treatment can reduce silica–silica interactions and promote silica–polymer interactions, producing a composite with well-dispersed mesoporous silica in the polysulfone matrix (Kim and Marand, 2008; Orbeci et al., 2017). The SEM images of the membranes with functionalized silica filler (Figure 9) revealed a good dispersion of the MCM-41-NH_2_ particles through the polymeric matrix as well for the 20 wt%— shown in Figures 9e,f—due to amino groups, which prevent the formation of hydrogen bonds.

**Figure 10:**
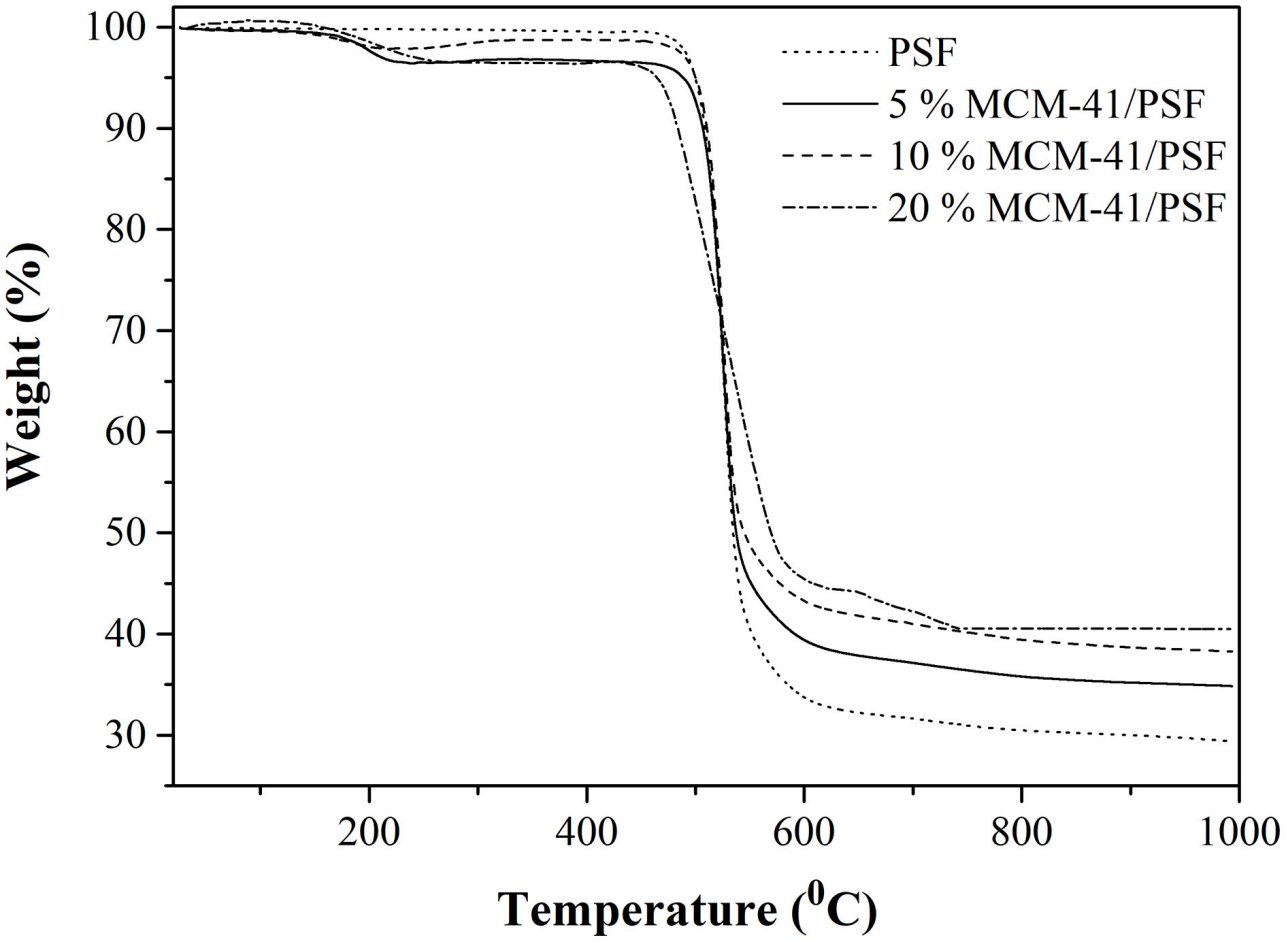
TG analysis of MCM-41/PSF.

From TG analysis of 5, 10 and 20 wt% MCM-41/PSF and neat PSF membrane (Figure 10), two notable weight losses were observed for all the samples. The first weight loss was around 200°C, due to trapped water and solvent molecules in the material. The second weight loss began at ~450°C and continued until the end of the analysis as a consequence of the polymer chain degradation. On the basis of the TG analysis, it can be stated that the residual content increased directly with the MCM-41 loading, from 29.37 to 40.87% of the total weight, confirming once again good dispersion of the mesoporous filler in the polymer matrix.

**Figure 11:**
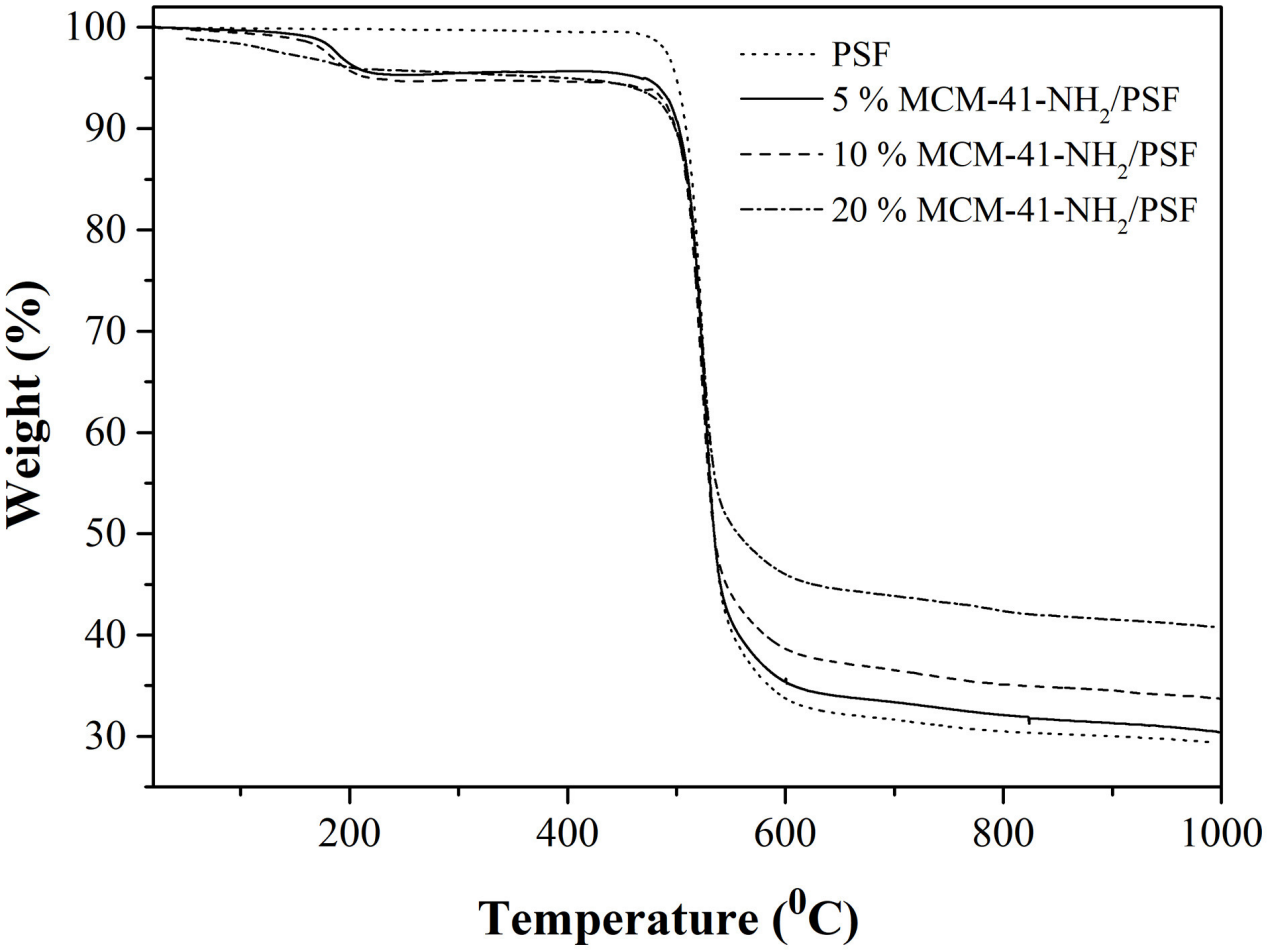
TG analysis of MCM-41-NH_2_/PSF.

In the case of the functionalized mesoporous silica-PSF membranes, the TG analysis (Figure 11) registered three mass loss steps: 30–220°C, due to trapped water and solvent molecules in the material; and 450–620°C, attributed to toluene residue and also to the amino groups' release. The mass loss continued above 620°C and resulted in the following residues: 32.47, 36.00, and 40.81% for 5, 10 and 20 wt% MCM-41-NH_2_/PSF, respectively.

Single gas permeability and the ideal selectivity values for the neat membrane and the mixed matrix membranes containing 5, 10 and 20 wt% MCM-41 or MCM-41-NH_2_ are presented in Tables 1, 2 respectively.

**Table 1:** Permeability of the mixed membranes.

**Membrane**	**Filler loading (wt%)**	**Membrane thickness (μm)**	**O_**2**_ (Barrer)**	**N_**2**_ (Barrer)**	**CO_**2**_ (Barrer)**
PSF	0	29	0.79 ± 0.05	0.09 ± 0.01	3.56 ± 0.09
MCM-41/PSF	5	30	1.21 ± 0.06	0.18 ± 0.01	4.56 ± 0.09
MCM-41-NH_2_/PSF	5	30	1.2 ± 0.06	0.16 ± 0.01	4.25 ± 0.09
MCM-41/PSF	10	30	1.55 ± 0.07	0.2 ± 0.01	6.45 ± 0.10
MCM-41-NH_2_/PSF	10	30	1.51 ± 0.07	0.18 ± 0.01	6.15 ± 0.10
MCM-41/PSF	20	31	1.78 ± 0.08	0.22 ± 0.01	8.08 ± 0.10
MCM-41-NH_2_/PSF	20	31	1.63 ± 0.07	0.19 ± 0.01	7.89 ± 0.10

**Table 2:** Selectivity of the mixed membranes.

**Membrane**	**Filler loading (wt%)**	**O_**2**_/N_**2**_**	**CO_**2**_/N_**2**_**
PSF	0	8.77	39.55
MCM-41	5	6.72	25.33
MCM-41-NH_2_	5	7.50	26.56
MCM-41	10	7.75	32.25
MCM-41-NH_2_	10	8.38	34.16
MCM-41	20	8.09	36.72
MCM-41-NH_2_	20	8.57	41.52

The values show a significant improvement in the permeability of the mixed matrix membranes (PSF with MCM-41 or MCM-41-NH_2_ filler) compared to the neat PSF membrane for all gases.

The introduction of the mesoporous material MCM-41 in the polymeric matrix had a positive effect, thus the permeability values increased directly with the silica quantity from the membrane (Table 1). In the case of O_2_, the permeability increased from 1.21 Barrer (for 5 wt% loading) to 1.78 Barrer (for 20 wt% loading). For N_2_, the increase was not significant, namely from 0.18 (for 5 wt% loading) to 0.22 Barrer (for 20 wt% loading). For CO_2_, an important increase was observed, almost double, from 4.56 Barrer (for 5 wt% loading) to 8.08 Barrer (for 20 wt% loading).

In the case of MCM-41-NH_2_/PSF, the O_2_, permeability increased from 1.20 Barrer (for 5 wt% loading) to 1.63 Barrer (for 20 wt% loading). For N_2_, the increase was not significant, namely from 0.16 (for 5 wt% loading) to 0.19 Barrer (for 20 wt% loading). Also in this case, for CO_2_, an important increase was observed, from 4.25 Barrer (5 wt% loading) to 7.89 Barrer (20 wt% loading).

The results obtained for MCM-41-NH_2_/PSF membranes indicated that N_2_ permeability was negligible (Table 1); however, they displayed higher CO_2_/N_2_ selectivity than the MCM-41/PSF membranes (Table 2).

The increase in permeability suggests that the penetration of the polymer chains would not block the mesoporosity of MCM-41. As the filler was replaced by MCM-41-NH_2_, the permeability slightly decreased. Also, the addition of mesoporous MCM-41 led to a small loss of selectivity for O_2_/N_2_. Analyzing the selectivity values of the membranes for CO_2_/N_2_, it was observed that the PSF membrane was more selective than the mixed matrix membranes. The selectivity loss is considered normal, taking into account that the PSF is a rigid polymer and, generally, the membranes obtained only from this polymer are dense, the gas transport being based on solution-diffusion mechanism. The mixed matrix membranes based on PSF and MCM-41 were more selective for CO_2_/N_2_ than for O_2_/N_2_ due to the small difference between O_2_ and N_2_ and the higher difference between CO_2_ and N_2_ molecule dimensions. Furthermore, the critical temperatures of CO_2_ and N_2_ are 31°C and−147.1°C, respectively. Taking into account this aspect, the solubility and the permeability of CO_2_ were expected to increase.

In the case of mixed matrix membranes based on PSF and mesoporous functionalized MCM-41, the permeability increased directly with the MCM-41-NH_2_ content in the membranes. Also, the mixed membranes obtained with MCM-41-NH_2_ filler were more selective than the PSF/MCM-41 membranes. The increase of the selectivity was mainly determined by the amino groups from the MCM-41 filler, leading to a stronger interaction of the two materials (organic polymer-mesoporous silica) and to the membranes stiffening.

**Figure 12:**
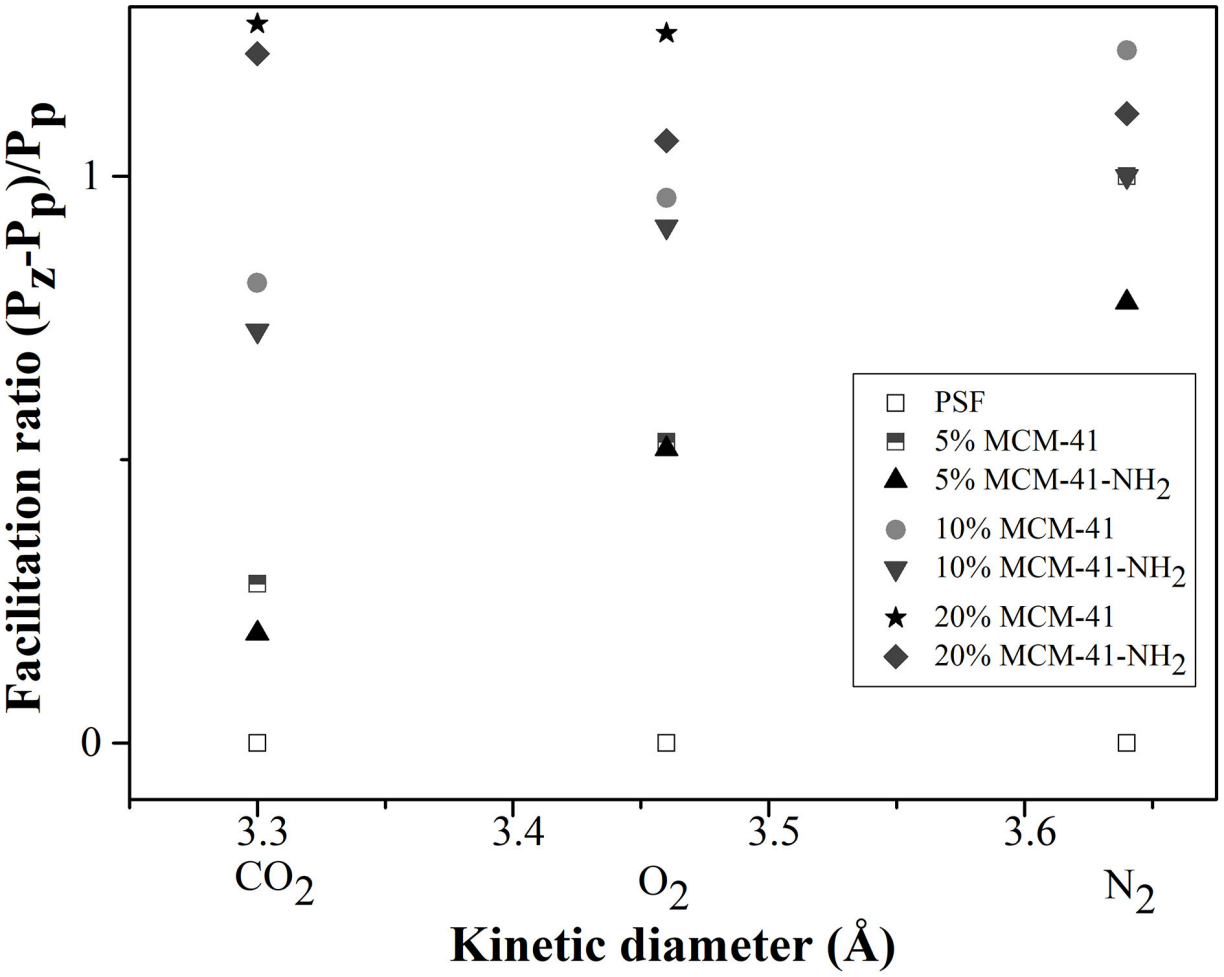
Facilitation plot of measured gas for MCM-41/PSF and MCM-41-NH_2_/PSF membranes.

Figure 12 shows the facilitation plot of the gases measured for mesoporous MCM-41/PSF and MCM-41-NH_2_/PSF membranes (5, 10, and 20 wt% loading).

The facilitation ratio is the difference in the permeabilities of the membrane and the pure polymer divided by the permeability of the pure polymer. At these loadings, the mesoporous materials essentially acted as fillers (Jomekian et al., 2012). It was noted that, at higher loadings, a channel network might form, connecting separated voids and allowing the rapid permeation of all gases. MCM-41/PSF membranes exhibited an immediate increase in permeability with adsorbent loading (from 6.72 Barrer for 5 wt% loading to 8.09 Barrer for 20 wt% loading). For MCM-41/PSF, the monotonic increase in permeability could be a consequence of the presence of mesopores within the MCM-41 framework rather than voids at the polymer/MCM-41 interface. When a gas molecule crosses over from the polymer phase into MCM-41 pores, it should encounter less resistance to flow as it is translated through the 40 Å wide channel, which is occupied by some measure of polymer (Reid et al., 2001).

**Figure 13:**
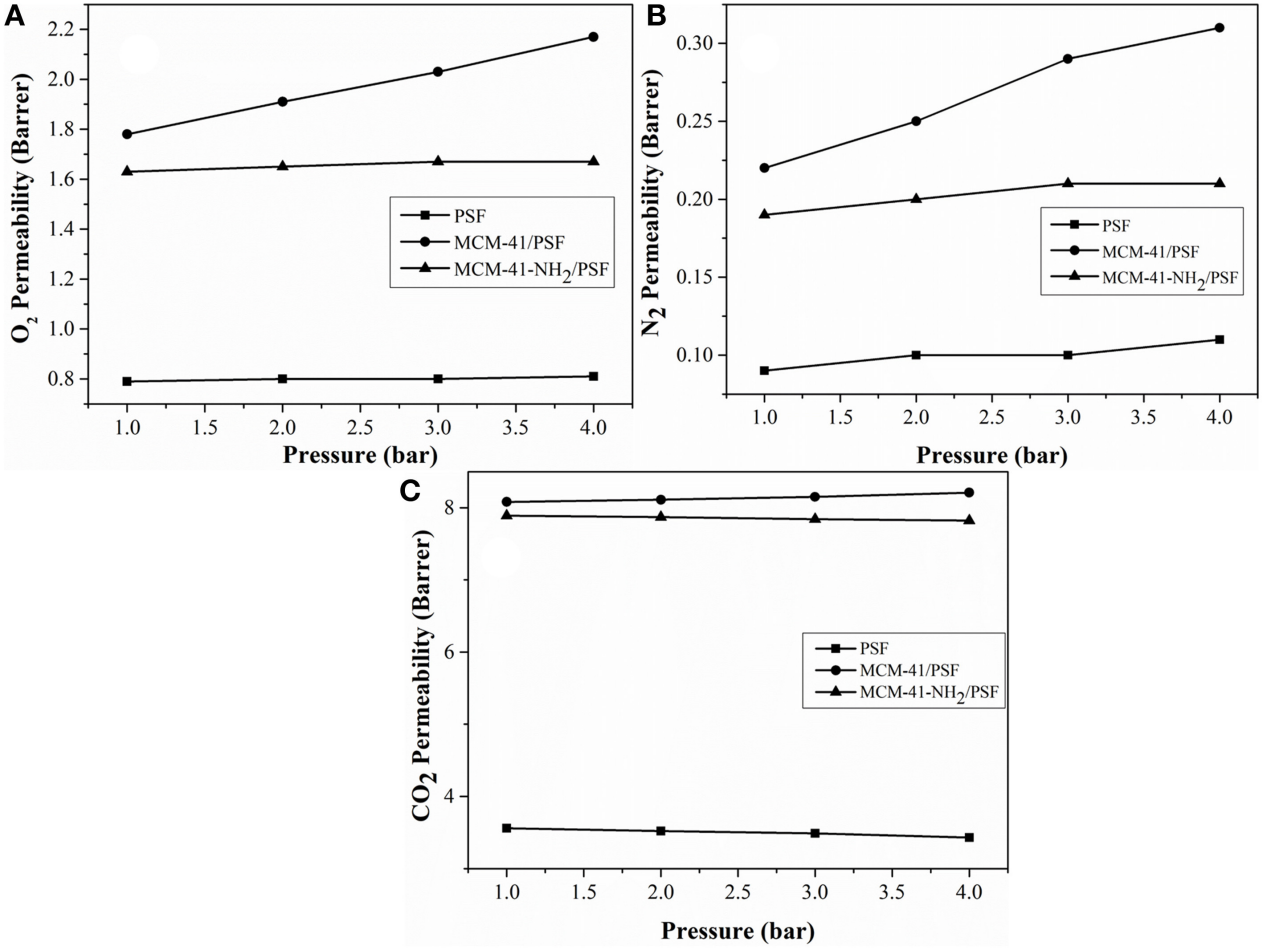
Pressure dependence of the permeability coefficient at ambient temperature. **(A)** O_2_ Permeability (Barrer); **(B)** N_2_ Permeability (Barrer); **(C)** CO_2_ Permeability (Barrer).

In order to confirm that the observed increases in permeability were due to the presence of non-selective voids at the MCM-41/PSF interface, the effect of varying the upstream pressure was investigated (Figure 13).

For the neat PSF membrane, O_2_ and N_2_ permeabilities were virtually independent of the driving pressure, while CO_2_ permeability slightly decreased as the pressure increased (Reid et al., 2001). If such non-selective passages exist in the mixed membranes, the change in pressure with respect to time on the downstream side of the membrane will be directly proportional to the driving pressure on the upstream side. In the case of the 20 wt% MCM-41/PSF membrane, the O_2_ permeability increased slightly, from 1.91 Barrer (1 bar) to 2.17 Barrer (4 bar), and the N_2_ permeability also increased slightly from 0.22 Barrer (1 bar) to 0.31 Barrer (4 bar). CO_2_ permeability increased from 8.08 Barrer (1 bar) to 8.21 Barrer (4 bar).

In the case of the 20 wt% MCM-41-NH_2_/PSF membrane, the O_2_ and N_2_ permeability remained almost constant, from 1.63 Barrer (1 bar) to 1.67 Barrer (4 bar), and from 0.19 Barrer (1 bar) to 0.21 Barrer (4 bar), respectively. CO_2_ permeability slightly decreased from 7.89 Barrer (1 bar) to 7.82 Barrer (4 bar), demonstrating that in this case, there are not any non-selective voids.

Excellent membrane performance requires both high selectivity and high permeance, according to Robeson's rule (Robeson, 2008). However, there is a trade-off between the two; a high loading of amine groups in the mesoporous material assures high selectivity, but low permeance values. On the other hand, high CO_2_ permeance with low amine loadings leads to lower selectivity. For example, some studies reported reverse selective properties wherein CO_2_ molecules were trapped and passed more slowly through the membrane than other gases when amine groups with very high affinities for CO_2_ were used (Kumar et al., 2008; Kim et al., 2015b). Therefore, cross-linking occurred and resulted in sticky diffusion of the CO_2_, due to the strong affinities of the amine groups. Thus, appropriate functionalization agents must be employed when developing separation-mixed matrix membranes.

## Conclusions

Mixed membranes for gas separation represent a rapidly growing research field for the porous materials community. A simple method to prepare mixed matrix membranes with mesoporous silica (obtained via the sol–gel method) was presented. The use of two mesoporous materials (ordered mesoporous silica MCM-41 and MCM-41-NH_2_) to produce mixed matrix membranes not only improved the filler dispersion and interaction into the polymer, as shown by the XRD, SEM, TG, but also gave rise to a significant enhancement in the separation performance. The tests showed that the mixed membranes had better gas separation properties than the neat PSF membrane under the same conditions. Results indicated that the mesoporous silica additive served to enhance the diffusivity and overall permeability of the small molecules without a loss of selectivity. The increased permeability resulted from the increase of the solubility and diffusivity. Amine-functionalized mesoporous membranes show significantly promising CO_2_ separation due to the strong adsorption properties of the surface amine groups and the regular mesoporous structure. Comparing the results with the up-to-date literature, it can be stated that, along with polymers, zeolites, metal organic frameworks, and mixed-matrix membranes with mesoporous silica as filler represent a technologically scalable platform.

## Author Contributions

MM conceived and designed the study, contributed to the collection of data, performed part of the analysis techniques and contributed to the data interpretation and to the manuscript writing. CI contributed to the data interpretation and to the manuscript writing. GN performed part of the analysis techniques and contributed to the data interpretation. V-CN contributed to the design of the study, supervised the project, performed part of the analysis techniques, contributed to the data interpretation and to the manuscript writing. All authors discussed the results and contributed to the final manuscript.

### Conflict of Interest Statement

The authors declare that the research was conducted in the absence of any commercial or financial relationships that could be construed as a potential conflict of interest.
